# When do Physicians and Nurses Start Communication about Advance Care Planning? A Qualitative Study at an Acute Care Hospital in Japan

**DOI:** 10.1007/s41649-020-00135-1

**Published:** 2020-07-13

**Authors:** Mari Tsuruwaka, Yoshiko Ikeguchi, Megumi Nakamura

**Affiliations:** 1 Division of Bioethics/Nursing Ethics, Graduate School of Nursing Sciences, St. Luke’s International University, Tokyo, Japan; 2 Division of Nursing, Musashino University, Tokyo, Japan; 3 People-Centered Care Research Department, St. Luke’s International University, Tokyo, Japan

**Keywords:** Advance care planning, Advance care planning communication, Acute care hospital, Japan, End of life care

## Abstract

Although advance care planning (ACP) can lead to more patient-centered care, the communication around it can be challenging in acute care hospitals, where saving a life or shortening hospitalization is important priorities. Our qualitative study in an acute care hospital in Japan revealed when specifically physicians and nurses start communication to facilitate ACP. Seven physicians and 19 nurses responded to an interview request, explaining when ACP communication was initiated with 32 patients aged 65 or older. Our qualitative approach employed descriptive analysis to identify major themes, which included “initiation by patients” and “initiation by healthcare professionals.” In the latter case, seven specific triggers were identified: (1) when the patients’ medical condition changed in terms of symptom relief, (2) when the patients’ medical condition changed in terms of prognostic prediction, (3) when serious events occurred, (4) when a choice of treatment was presented, (5) when the location for end-of-life care was chosen, (6) when the patients’ cognitive function deteriorated, and (7) when serious events settled down. Within this group of healthcare professionals, physicians were more focused on changes in their patients’ medical condition, whereas nurses focused more on their patients’ desire for a long-term perspective. Nurses encouraged patients to consider ACP themselves, which developed into an approach to respect patients’ autonomy. In acute care hospitals, it appeared to be desirable to have an early discussion where patients could understand the significance ACP, which would matter even after their discharge from the hospital.

## Background

Advance care planning (ACP) has been conceptualized on the ethical basis of respect for patients’ autonomy (Johnstone and Kanitsaki 2009; Fahner et al. 2018) and is expected to improve the quality of medical treatment and care and realize the goal of patient-centered care. It has been defined as the process through which patients discuss their wishes and preferences regarding their medical treatment, end of life care, and often also the location of those, with their family, those dear to them, and medical staff, in advance of a potential decline in mental capacity. ACP communication is particularly important in acute care hospitals, where several challenges have been identified, including the following five.

First, lifesaving medical care is often prioritized over ACP communication (Tan et al. 2019). While ACP is usually documented with a written advance directive (AD), the emphasis of the process is on communication. Previous ACP surveys in acute care hospitals have nevertheless primarily examined the completion rate of advance care plans and ADs in written form, through retrospective review of medical records and comparative study of in- and outpatient environments (Malyon et al. 2017; Peck et al. 2018; Schofield et al. 2015; Scott et al. 2016). In other words, most of these studies tended to focus on AD completion and few studies in acute care hospitals have focused on the communication process (Solis et al. 2018).

Second, the short length of hospitalization and consultation in acute care hospitals does not allow long and frequent discussion with patients. (Hynes et al. 2015; Scott et al. 2016; Dixon and Knapp 2018; Rogers et al. 2019; Meehan et al. 2019). In this context, it has to be noted that ACP communication can be time consuming. Therefore, a lack of human resources has also been indicated as important (Tan et al. 2019).

Third, it is often not clear who should take the initiative to discuss ACP (Meehan et al. 2019; Spence et al. 2009). Some may perceive that higher education levels and medical training place physicians in a position of authority and that ACP communication should therefore follow their treatment recommendations (Nedjat-Haiem et al. 2017). Of course, patients need to know their treatment options. Therefore, the patient’s preferences need to be considered in concert with physicians (Johnson et al. 2016; Hajizadeh et al. 2014). There is no agreement, however, or not even much discussion, on who should take the initiative in ACP communication (Vanderhaeghen et al. 2018). Although many research papers emphasize the role of general practitioners (GP) (Howard et al. 2018; Solis et al. 2018), there is insufficient ACP collaboration between GPs and hospital doctors (Wichmann et al. 2018) and communication or agreement in care among physicians, more generally (Fanta and Tyler 2017). It is pointed out that in acute care hospitals, information about patients’ ACP is often not transferred from one ward to another or to healthcare providers involved in homecare (Johnson et al. 2016; Tan et al. 2019).

Fourth, patients and families are not always ready to consider ACP (Rhee et al. 2012; Smith et al. 2014; Meehan et al. 2019). Most patients in acute care hospitals hope their medical treatment will result in recovery and are not always willing to discuss their prognosis and outlook (Wichmann et al. 2018; Zwakman et al. 2018).

Finally, with respect to the best moment for starting ACP communication, when an individual’s medical condition is worsening, it should occur sooner rather than later (Epstein et al. 2015; Bakitas et al. 2016). Several surveys on a multidisciplinary basis indicated that ACP communication should be started when the patient’s chances for recovery are declining (Vanderhaeghen et al. 2018); when an individual’s health condition worsens; when there is a fear of losing capacity; and when aging progresses (Rietjens et al. 2017; Wichmann et al. 2018).

In fact, in spite of these challenges, hospitalization can be a catalyst that increases the perceived relevance of ACP for some patients (Knight et al. 2020; Peck et al. 2018). ACP communication in acute care hospitals, and when to start it, is therefore important for patients.

Spreading internationally, the concept of ACP was incorporated in Japan as one of the notable revisions to the “Guidelines on the Decision Process of Healthcare at the Stage End of Life” (Ministry of Health, Labor and Welfare 2018). These guidelines now emphasize the importance of approaches to ACP, in which such subjects as healthcare planning and what life an individual wishes to lead, should be discussed repeatedly among patients, their family, those they trust, and their healthcare team, in response to their physical and mental changes and the subsequent changes in their intention (Ministry of Health, Labor and Welfare 2018).

In Japan, empirical ACP research has been carried out primarily in palliative care and home care settings (Ishikawa et al. 2017; Naito et al. 2016). The authors of this paper, for example, identified in a previous study in 2016, six occasions when home care nurses start ACP communication with the elderly: (1) upon commencing home care, (2) while providing daily care, (3) upon changes in the health condition, (4) during the terminal phase, (5) when the nursing care burden on family members becomes overwhelming, and (6) when the nursing requirements of the patient have exceeded levels that can be managed by family members (Tsuruwaka et al. 2016). These were concerned with daily care, the prospect for the future, urgent need for decision-making, and various other situations. It was also revealed that ACP communication was performed repeatedly even in the same case. Nurses, in particular, drew out the elderly’s intentions from what sentiments were expressed in their daily conversation while they were receiving care, as well as from their mental and physical changes.

Since GPs are not a well-accepted type of medical provider in Japan, ACP communication is mostly started in acute care hospitals. Since the timing at which physicians and nurses hold ACP communication has a major impact on patients due to the short length of their hospital stay, it is extremely important to identify this timing. ACP is of importance for individuals of every age, particularly for the elderly from a perspective of end-of-life care.

This study aimed to present when physicians and nurses in acute care hospitals in Japan start ACP communication using case studies of elderly patients, in order to help facilitate practices of ACP.

## Methods

### Study Design

A qualitative descriptive approach (Sandelowski 2000) using 60-min semi-structured interviews in Japanese was used to study ACP communication with patients aged 65 or older. The interviews discussed the following: (1) a case of an elderly patient who they talked with about ACP, (2) when they started talking about ACP with that particular patient, and (3) the difficulties in timing ACP communication.

### Data Collection Method

This survey was conducted in one acute care hospital in Japan from October 2017 to March 2018. The participants were physicians and nurses with 5 years or more of experience, as this allowed them to talk about ACP communication from a broader perspective with more objectivity and impartiality. The selection of participants was further based on three criteria: (1) they were involved with patients who were 65 years or older, (2) these patients could communicate at the start of involvement, and (3) they discussed these patients’ intentions and wishes regarding their treatment and care.

There were two reasons why we asked participants to talk about a specific patient aged 65 or older. First, because this demographic is significant among patients in acute care hospitals in Japan as society ages rapidly at a rate of 28%. Second, because when patients of 65 and above are discharged from an acute care hospital, they are referred to a different type of hospital, a nursing home, or a return home. In addition to the continued need of ACP in each of these care settings, the involvement of acute care healthcare professionals in ACP is seen as authoritative, also in these later care settings.

### Data Analysis

We repeatedly read the verbatim record according to the context, focusing on when physicians and nurses thought they should initiate ACP communication. We extracted some specific contexts to define and name some codes that represented the main meaning of the description. The codes were divided by similarity of meaning and redefined as a few decades of categories. After the categories were compared with each other and classified according to the similarity and correlation of the contents, several categories were defined with the degree of extraction increased. Newly added data was similarly compared with the existing categories and examined regarding how they were related with each other, with the degree of extraction increased to clarify the initiation of ACP communication. In addition, we compared the similarities and differences in the initiation of ACP communication between physicians and nurses. We repeatedly discussed data collection and analysis to ensure the adequacy of coding.

### Ethical Consideration

We explained the research content to the head ward administrator and presented him with the document and asked him to give it to the staff to have them distribute some copies and to post it on the bulletin board. Interested physicians and nurses were to directly contact us. All participants received a written explanation of the study’s purpose, methods, and confidentiality requirements, and we emphasized the voluntary nature of participation and their right to withdraw at any time. Regarding confidentiality, interviews were recorded, and notes were anonymized and stored securely in a locker to be kept on record for at least 5 years after publication in an academic journal. After that time, all records will be destroyed. The study was conducted after obtaining approval from the Institutional Review Board (IRB) of St. Luke’s International University (No. 17-A046).

## Results

### Basic Attributes of Subjects

Twenty-six subjects were composed of seven physicians and 19 nurses and their attributes are shown in Table 1. Among them, there were 20 women and 6 men with an average of 23.4 years of experience. Table 2 includes the overview of 32 case patients. The details of the cases, 18 female patients and 14 male patients mostly with cancer, are indicated in Table 3. As presented in Table 3, two cases from one nurse, three cases from one physician, and two cases per person from three physicians were provided. As shown in Table 4, the period of time the participants were involved with the patients ranged from 6 days or less to 10 years or more, with 1 month to less than 6 months being the most common. The settings in which these subjects communicated with their patients were eight cases of outpatient clinic visits and an inpatient ward, and 12 cases of outpatient clinic visits only, all attended by the physicians; and 8 cases of outpatient clinic visits, 11 cases of an inpatient ward, and 1 case of a consultation support center, all attended by the nurses.

**Table 1:** Participant characteristics

Characteristics of interviewee			Number of participants
Profession		Physician	7
		Nurse	19
Sex		Male	6
		Female	20
Number of years of experience		0–5	0
		6–10	3
		11–15	12
		16–20	14
		21–25	3
		26–29	1
		30-	3
Mean years of experience			23.4
Physician	Job title	Director	3
		Deputy director	2
		Head medical staff	1
		Staff	1
	Specialty	Oncology	2
		Cardiology	1
		Hematology	1
		Palliative care	1
		Nephrology	1
		Endocrinology	1
Nurse	Job title	Nurse manager	2
		Assistant manager	6
		Staff	11
	Advanced certification	Certified nurse specialist (cancer nursing)	3
		Certified nurse specialist (chronic care nursing)	1
		Certified nurse (chronic heart failure nursing)	1
		Certified nurse (palliative care)	1
		Certified nurse (intensive care)	1
		Certified nurse (dementia nursing)	1
		Certified nurse (dialysis nursing)	1
	Department	Outpatient	5
		Inpatient ward	7
		Palliative care unit	2
		Intensive care unit	1
		Kidney center	1
		Social service center	3

**Table 2:** Case characteristics (*n* = 32)

Case characteristics		Number of respondents
Sex	Male	14
	Female	18
Age group	60s	13
	70s	6
	80s	10
	90s	3
Disease	Cancer	17
	Cardiac arrest	4
	Chronic renal failure	3
	Myelodysplastic syndromes	2
	Cerebral infarction	1
	Arteriosclerosis obliterans	1
	Amyotrophic lateral sclerosis	1
	Fracture	1
	Diabetes	1
	Spine injury	1

**Table 3:** Case profiles (*n* = 32)

	Health providers No.	Patients No.	Age-group	Sex	Disease
Nurse	1	001	80s	F	Cardiac arrest
	2	002	90s	F	Cardiac arrest
	3	003–1	60s	M	Chronic renal failure
		003–2	60s	F	Chronic renal failure
	4	004	60s	M	Amyotrophic lateral sclerosis
	5	005	70s	M	Esophageal cancer
	6	006	70s	F	Endometrial cancer
	7	007	80s	F	Breast cancer
	8	008	70s	M	Cerebral infarction
	9	009	60s	F	Breast cancer
	10	010	80s	F	Pancreatic cancer
	11	011	60s	F	Ovarian cancer
	12	012	80s	F	Breast cancer
	13	013	80s	F	Cecum cancer
	14	014	70s	M	Spine injury
	15	015	60s	M	Prostate cancer
	16	016	70s	F	Stomach cancer
	17	017	80s	F	Fracture
	18	018	60s	F	Ovarian cancer
	19	019	60s	M	Cardiac arrest
Physician	20	020–1	80s	F	Malignant myeloma
		020–2	90s	F	Myelodysplastic syndrome
		020–3	70s	M	Myelodysplastic syndrome
	21	021	80s	M	Lung cancer
	22	022–1	60s	F	Breast cancer
		022–2	80s	F	Pancreatic cancer
	23	023–1	60s	M	Cardiac arrest
		023–2	60s	M	Arteriosclerosis obliterans
	24	024	60s	M	Ureteral cancer
	25	025	80s	M	Chronic renal failure
	26	026–1	60s	F	Colon cancer
		026–2	90s	M	Diabetes

**Table 4:** Participants’ interactions with 32 patients

Period of time	No. of patients
6 days or less	1
1 week–less than 2 weeks	2
2 weeks–less than 1 month	4
1 month–less than 6 months	5
6 months–less than 1 year	4
1 year–less than 3 years	3
3 years–less than 5 years	2
5 years–less than 10 years	7
10 years or more	1
N/A	3

### Initiation and Triggers of ACP Communication

Subjects determined that ACP communication was sometimes initiated by patients and sometimes initiated by healthcare professionals. In the first case, it did not mean that patients explicitly called for ACP, but that they mentioned a thought that could be construed to develop into ACP. In the second case, when healthcare professionals initiated ACP communication, seven triggers could be identified: (1) when patients’ medical condition changed in terms of symptom relief, (2) when patients’ medical conditions changed in terms of prognostic prediction, (3) when serious events occurred, (4) when a choice of treatment was presented, (5) when the location for end-of-life care was chosen, (6) when the patients’ cognitive function deteriorated, and (7) when serious events settled down. Specific narratives of each trigger are shown below. “D” in parentheses represents a physician, “N” a nurse, followed by a case number.

#### The Patients’ Initiative

As patients communicated their thoughts leading to ACP to healthcare professionals, these healthcare professionals offered suggestions and support to help them think about ACP as follows.

A woman in her nineties with myelodysplastic syndrome (MDS) underwent blood transfusion as an outpatient instead of anticancer drugs to deal with anemia. Her physician judged from her condition that the prognosis was poor and that what to do in the future should be decided. Her thoughts presented below prompted him to help her create a living will.

She said repeatedly, “I don't want to live further because I've lived long enough. I don't want to be hospitalized. I'd prefer coming to the hospital for blood transfusion.” Most people are wishing treatment reduced, they don't say so though. But she is telling me this again and again. I handed a booklet of living will over to her son, who always came with her. He knew her condition and I had talked about her illness. I advised him to talk with her and confirm what she would wish when she became seriously ill. (D/020-2)

At the stage where anticancer drug treatment was no longer effective, a man in his seventies with myelodysplastic syndrome (MDS) asked to be shifted to home therapy. On regular visits, he explained how he was thinking about therapy to extend his life. As in the previous case, this was taken as an opportunity to suggest considering a living will.

He said, “I'm happy because I've lived long enough up to over seventy years of age despite this disease.” It is often the case that this subject appears for a moment in a daily conversation regardless of context. "When the end has come, I don't want to do what I can no longer bear," or "How long can I be alive?" When these kinds of words come out, as seen in his case, I take it as the chance to discuss. (D / 020-3)

A patient in her eighties with stage IV breast cancer was not able to say straightforwardly what she wanted to tell her doctor.

Her only one wish was to spend time with peace and no pain, having fun with other people. Around the evening, I found her complaining of another strong pain. She looked really distressed. I was listening to her when she suddenly murmured the words. (N / 012)

The nurse respected her words and helped her tell her doctor of her thoughts, being together at the next doctor visit, which resulted in considering what care would be needed after she was discharged from the hospital.

In other instances, healthcare professionals approached patients to initiate ACP communication. In these circumstances, seven different triggers were identified, numbered below from 2 to 8.

#### When the Patients’ Medical Condition Changed in Terms of Symptom Relief

As no further treatment was found for a patient in her sixties with breast cancer, the next possible phase to shift into was palliative care. She had an aged husband, who lived in a remote place. Where she would receive end-of-life care to alleviate her condition had to be discussed early on with her family. She was prompted to think about ACP, including palliative medical care as well as the place for end-of-life care, i.e., at a palliative care department.

Patients are referred to the palliative care unit ward when all treatment ends. Just before the last anti-cancer drug is used, we begin to think about the next phase and go with the collaboration of palliative care. If we decide to shift to it, I’ll attend the consultation and suggest thinking about various future options. (N / 009)

#### When the Patients’ Medical Condition Changed in Terms of Prognostic Prediction

The doctor assumed that the prognosis of a woman in her eighties with pancreatic cancer was severe at the time of the first consultation. She was prompted to discuss her future prospects early and consider ACP.

At the time of her hospitalization, we realized she was really entering the terminal phase from the progress of her cancer and the spread to the liver. At the timing of her hospitalization, I talked about ACP so that she could get ready for the end of life. (D / 022-1)

A woman in her seventies with gynecological cancer was aware of her illness at the time of hospitalization, but was not told about her prognosis, due to her husband’s strong request. The nurse knew that the end of her life would come soon and that her wishes would not be fulfilled. After the nurse consulted with her husband many times and talked with her as follows, she was finally able to talk with her family.

I asked if there was something she wanted to tell, leave and do for her husband and family. She was unable to get out of the bed at all, but she was fully conscious and could communicate with others. I made up my mind to talk about ACP right then to her, finding her health status narrowly maintained. If I had missed the moment, she would have become too ill to have a chance to talk. I thought, “Now is the time. This is the last chance.” (N / 006)

#### When Serious Events Occurred

Serious events could, for example, include a sudden seizure.

In the case of a man in his sixties under observation as an outpatient after cardiac infarction, the physician believed that the occurrence of serious events was a good time to consider ACP.

He entered the ICU with his heart status worsened and severe pneumonia. While he was isolated from others, I found it a little difficult for him to make decisions on his own and asked him if he wanted to undergo chemotherapy as another means. There were several things to discuss and decide about his future, such as a choice between resuscitation and DNAR and how to respond to some sudden changes. When serious events occurred is a good and accessible timing for him and his family to think about his future. (D / 023-2)

A woman in her eighties with malignant myeloma had been well without pain, but her eyes suddenly got worse. As no abnormalities in her eyes were identified, despite many examinations, it was concluded that her condition was caused by worsening myeloma. This event became an opportunity to ask her about what she would wish for in the future.

She lost her sight and developed cell tumor. I explained to her it was no longer normal and there was no treatment we could do for her to recover. I told her that myeloma was getting worse and the time was about to come. I usually avoid talking about death, but was able to talk frankly because she was over eighty years old and didn't look like getting upset easily. I asked what she would wish to do in the future. (D / 020-1)

#### When a Choice of Treatment Is Presented

A man in his sixties with urinary cancer did not want to have a serious conversation and tried skipping it. A choice among treatment options was used as an opportunity to try and prompt him to think about ACP.

The anti-cancer drug worked for about one month only. I saw his legs badly swollen and the pain in the lower back got worse. I suggested shifting to the treatment for the second stage right away. When he needed to choose among treatment options, I asked little by little how he wanted to live. He told me that he wanted to work home, which was important for him. (D / 024)

#### When the Location for End-of-Life Care Was Chosen

A woman in her eighties with gastrointestinal cancer, who was supposed to be discharged from an acute care hospital, was prompted to think about the next place for undergoing treatment.

She had not been diagnosed with dementia, but I noticed her declining cognitive function. Because normal cognition still remained, I wanted to talk with her to make sure of her thoughts. As she was gradually getting to eat more food, I thought about when she would be discharged. Is her house equipped enough for her end-of-life care? Should home care be included in her end-of-life care planning? As we talked about these kinds of social support, I asked her where she wanted to spend her terminal period. (N / 013)

#### When the Patients’ Cognitive Function Deteriorated

A woman in her eighties, who was hospitalized due to a fracture and assumed to have her cognitive function further declining, was prompted to think about her intentions and future.

She was diagnosed with dementia at a nearby clinic due to her cognitive ability gradually declining. But I found her clearly express her ideas when I asked her in a way accessible to her. Even though she would have to enter a nursing home, I thought I must talk and make sure how she was feeling. Because her cognitive function dropped considerably, it took a long time to ask her several questions. I found it difficult for her to answer them properly even if I tried to get her replies in a rush. (N / 017)

#### When Serious Events Settled Down

With serious events settling down, patients started to recover.

A woman in her eighties presented at the nursing department for outpatients with heart failure. When recovered, she was provided with an opportunity to think about what to do when something similar were to happen again and to think about ACP.

In most cases, palliative care for patients with heart failure used to be launched after they were hospitalized and fell into a difficult situation. I start talking with them when their condition begins to get worse and ask at first general questions such as what they think is important for them, not urging them to make a final decision. She tried to be cautious to avoid surgery and hospitalization but fell into a bad condition. When I asked her what she was going to do if she could not do without medical devices such as a respirator, she wasn’t able to answer due to something indefinite held in mind. Since she recovered from a serious event, she has been thinking about her terminal care with me every time she comes to see me for examination. (N / 001)

A woman in her nineties with chronic heart failure and her family were, given her old age, prompted to think about her living will when her condition became stable after the event.

She had recovered from heart failure and was discharged. She was spending a while in somewhat good health and her long stable condition made me become hesitant to begin the conversation about her terminal care. I asked her family to decide early when she got better, because she wouldn't be able to decide anything when distressed. I asked what they were thinking in seeing her getting older and gradually more ill. I explained about a living will and handed out a booklet to talk again at the next hospital visit and asked them to talk with her at a good occasion. (N / 002)

### The Difference Between Physicians and Nurses

While physicians tended to start ACP communication “when a choice of treatment was presented” and “when serious events occurred,” nurses started ACP communication “when the patients’ cognitive function deteriorated” and “when serious events settled down,” which was not observed with physicians.

## Discussion

This survey indicated that there were two major types of initiation of ACP communication: “initiation by patients” and “initiation by healthcare professionals.” Which type of initiation of ACP communication should be recommended as the principle one in acute care hospitals? From an ethical standpoint, it appeared evident that “when patients expressed their thoughts leading to ACP” should not be missed in any setting, not to mention in acute care hospitals. ACP communication should begin once patients show their intention in any form. Previous research also determined that ACP communication should be initiated with some implicit intentions of patients (Johnson et al. 2017; Lum et al. 2016). In other words, ACP communication that patients do not wish or that is led and moved forward by healthcare professionals can be paternalistic. However, patients often do not realize when they should start ACP communication because they cannot fully imagine their prognosis and possible future situations. Starting ACP early enough can be difficult for patients as ACP is often experienced as conflicting with hope for a cure (Wichmann et al. 2018). The practice of ACP particularly requires an accurate description of disease, the feasibility of treatment, and the description of future possible incidents, and patients’ understanding of these (Rietjens et al. 2017). Introducing ACP as soon as possible when any diagnosis is presented (Brown et al. 2012) and encouraging patients to consider the value of thinking about ACP, even in acute care hospitals and after discharge from the hospital, will lead to timely care.

As presented in the results, seven triggers determined by healthcare professionals were identified. In terms of such triggers, previous studies indicated that healthcare professionals make decisions when an individual’s health condition worsens and there is a fear of losing capacity in the near future (Rietjens et al. 2017; Wichmann et al. 2018; Vanderhaeghen et al. 2018). In the authors’ 2016 survey on ACP in a home care setting in Japan, the trigger for preparing for deterioration of mental and physical functions and capacity was similarly identified. Also, five triggers in the current study were exactly the same as those identified in the previous one: “change in patients’ medical condition in terms of symptom relief,” “change in patients’ medical condition in terms of prognostic prediction,” “when serious events occurred,” “choice of treatment,” and “deterioration of cognitive function”. According to healthcare professionals’ narratives, as shown in the results, their approaches tended to be performed in a situation where patients’ quality of life improvement resulting from treatment entered a difficult phase. Major changes in patients’ mental and physical conditions, in particular, caused triggers for ACP communication. In other words, a patients’ worsening health condition focused attention. The concept of ACP calls for early discussions before their ill health becomes serious. It is argued that future health uncertainties will become a strong motivation for patients to consider ACP (Peck et al. 2018). It is overdue to start a conversation after cognitive decline is observed. Starting a conversation earlier when such a decline is anticipated would benefit patients.

In terms of difference in triggers between physicians and nurses, physicians initiated ACP communication primarily “when a choice of treatment is presented” and “when serious events occurred.” Meanwhile, nurses were focused on their patients’ daily life, with close attention to concerns about the location for end-of-life care and wishes for their future life. In addition, the nurses regarded the first outpatient clinic visit and interview after serious events as an important occasion to discuss. There were both similarities and differences in approaches to patients between the physicians and nurses. Considering that physicians are responsible for the entire treatment of patients, and nurses plan their care in terms of their life, the characteristics of the two kinds of timing are understandable. Important elements of ACP are “not documentation of an advance directive but communication” and “not treatment preferences but patients’ values.” (Sudore et al. 2017) Since ACP emphasizes preparation for the future rather than current decisions (Randall 2011), it is important to learn the patients’ values and preferences. This survey revealed that physicians tended to discuss limited areas of treatment options according to the content of AD as patients’ diseases progressed. It is said that physicians’ discussions on ACP are not sufficient in terms of value-based discussions (Dixon and Knapp 2018), and our study, comparing physicians with nurses, also came to the same conclusion. It is desirable to discuss ACP continuously in daily clinical practice (Vanderhaeghen et al. 2018). Patient-centered approach taken by nurses in terms of their life and values is consistent with ACP’s goal of patients thinking in advance about medical and nursing care in light of their values. In acute care hospitals, most patients repeat outpatient clinic visits and hospitalization and thereby the department in charge changes depending on the health issue, which results in a disadvantage in which individuals involved with the patients change from time to time. In acute care hospitals, therefore, healthcare professionals need to share and give feedback on their interactions with patients with attention to ACP communication performed within the team.

Among the triggers for healthcare professionals to approach patients, found in this survey, one that was found only in the nurses’ approach was the trigger “when serious events settled down”. This trigger was obviously different from those associated with worsening health conditions. Interviews with nurses represented triggers when patients pondered what was significantly valuable for them, and what they should prepare themselves for from then on, looking back on events, and draw conclusions regarding their future plans.

The principle of respect for autonomy has been argued to be the ethical basis of ACP (Johnstone and Kanitsaki 2009; Fahner et al. 2018). Therefore, the ACP communication process itself can strengthen patients’ autonomous motivations (Lin et al. 2019). The triggers for nurses represented an approach to raise autonomy, in that patients could understand their condition and what could happen in the future, and subsequently think about their ACP. Seal (2007) stated that the process of discussing ACP is one of the roles of patient advocacy offered by nurses. The trigger of “when serious events settled down” appeared to be an approach that empowered patients and led to patient-centered care.

Since this study surveyed only one acute care hospital in Japan with many highly qualified professional staff, a more general theory cannot be drawn from the results. Further study on triggers of ACP communication in various hospitals is needed. In order to reveal triggers of ACP communication through interviews on a multidisciplinary basis, with various individuals who are involved in one particular case, it is necessary to scrutinize what sort of continual support is provided in the hospital, independent from a potential change of department in charge of the patient.

## Conclusion

This study identified that when physicians and nurses decided to start ACP communication, there were two major types: “initiation by patients” and “initiation by healthcare professionals.” In the latter case, there were seven different triggers determined. Physicians started ACP communication mostly “when the patients’ medical condition changed” and “when there was a choice of treatment;” in both cases, the effect of treatment could hardly be predicted. Meanwhile, nurses started ACP communication as a recommendation to consider future ways of life from a long-term perspective; this encouraged patients to think about ACP in the light of their own values. When communication about ACP in acute care hospitals starts, it can have important consequences, even after their discharge from the hospital.
